# Dr. Wood's Case of Confluent Small-Pox

**Published:** 1806-06

**Authors:** J. Wood

**Affiliations:** Newcastle


### *To the Editors of the Medical and Physical Journal.*

GENTLEMEN,

SINCE the Cow-pox engaged the attention of the medical world, as the means of preventing the horrors of the Small-pox, I fear little attention has been paid in a *direct* way to lessen those horrors when they *have* occurred.

While the friends of Vaccination have reared it from infancy to puberty, and given it a place in their own confidence and in that of the public, from which I trust it will never be removed; and while its enemies have been employed in throwing their arrows at its engaging form, to injure or destroy its growth, or while others have been engaged in free and liberal discussion of its merits, I believe *all* have omitted to suggest any improvement in the means of lessening the miseries and danger of the small-pox.

Now that the mind is relieved from all anxiety about the fate of the cow-pox, it will naturally turn itself to the consideration of the means of lessening the fatality and the pains of the small-pox, while unhappily any objects are left for it to seize; for I am sure every one of common humanity, who has witnessed any of the miseries and the fatality of the small-pox during the last year, could not but feel the desire of combating the disease in a more effectual manner than it is at present. In the true spirit of Christianity too, the friends of vaccination will feel equal pity for the prejudiced and the deluded, as for the ignorant, and will commiserate the fate of the victims of either, when suffering from the effects of the small-pox; thus with one hand presenting the cow-pock as a *shield*, and with the other lessening the pains of the small-pox in those who have been so unfortunate as not to be protected by it.

Influenced by such feelings, I beg leave to bring forward to notice a case of *confluent* small-pox, successfully treated by me some years ago, but by means, I believe, *very different* from what is generally thought requisite in *that species* of small-pox. In the *distinct* species there has long been no difference of opinion *amongst medical men*, as to the proper mode of treatment; but I have reason to know that in this place, and I dare say in many others, the treatment of the *confluent* species is, in general, very different from what I think it ought to be. It is, prima facie, indeed indeed strange, that any line should be attempted to be drawn between small-pox distinct or confluent, and more so that any particular mode of treatment should *stop* at such supposed line, and that *then* an opposite mode of cure should be thought necessary.

The case I am going to give you was under my care in the Infirmary in this town. It is but very seldom that a physician here has an opportunity of attending cases of small-pox in such a way as to be able to carry into full effect any particular mode of treatment, for in the middle and higher circles of society a case of casual small-pox never almost occurs, and amongst the poor, he finds neither the power nor the inclination to fulfil his views; amongst them he still finds the same attachment to heat from a warm fire, from blankets and confined beds, and the same aversion to cold air or cold water; even amongst nurses, and those who ought to be better informed, the idea still prevails, that washing and change of linen weakens and reduces the body; it is seldom therefore, and only in an Hospital, that the result of any particular mode of treatment in small-pox can be fairly ascertained.

It is now about seven years since the following case came under my care; and when the means of cure I attempted proved successful, I reserved it for another object,* besides that of recommending a more successful method of treating confluent small-pox: but I give it abstracted from every other view *at present* than that, which I most anxiously desire, of lessening the misery and mortality occasioned by the small-pox; and for this reason I will too refrain from making any remarks, but for the sake of explanation, on the means of cure I adopted.

Jan. 10, 1799, William Clark, aged 21, was admitted into the Infirmary on account of symptoms resembling those of acute rheumatism. The physician who received him, ordered the following medicine for him.

Antimon. tartar. gr. ¼; solut. omni hora donec purgat. deinde; pulv. cort. Peruv. ʒß ex infus. pectoral. 3tia. quaque hor.

The next day, the 11th, the physician who received him being at a distance, I was called upon to attend him, on account of an eruption that had appeared, which covered his body, and seemed to be small-pox. On inquiry, he said, that on the quay-side about fourteen days before,

* This, with your permission, I propose to enlarge upon; and, probably, in your next Number.

fore, he passed a woman with a child that had small-pox, and though at some yards distant from the object, he felt immediately a very *sickening* smell; he was a sailor and in full habit of body. He was now very feverish; I ordered him to be immediately taken to an out-building, which was kept ready to receive contagious diseases, to be well washed with cold water, and directed his medicines as follow.

Continuatur antimon. tartar. gr. $\frac{1}{4}$; solut. 2d q. q. hor. et capiat decoct. nitros. (sal nitri ʒj in decoct. hord. ℔j. solut.) pro potu. His diet was limited to gruel and milk.

14th, Eruption quite general, and already very confluent; fever less.

℞. Infus. senn. tartar. ℥ij. Kali tartar. ʒij ft. haustus mane quotidie sumendus. Continuatur decoct. nitros. ad ℔ij. per diem. Omittatur solutio, antimon. tartar.

16th. Fever much abated; symptoms favourable.

Continuatur decoct. nitros. et repetatur haustus purgans mane.

17th. From the 11th, his diet had been oatmeal gruel or milk. The purgative draughts operated always three or four times; he lay on a bed lightly covered with linen only; the season of the year was cool; a current of air passed constantly by his bed between an open door and window, and he was regularly washed; but as maturation took place, the washing was confined to his face and hands; his pulse was now under 100, his breathing not affected, no delirium; and though every part of his body was covered with a continued *sheet of matter*, yet he was not heated nor uneasy, and the swelling usual in distinct small-pox had taken place. Considering the maturation of the eruption now *at its height*, and dreading a secondary fever and other bad consequences from the absorption of so loose a quantity of morbific matter as must take place, I determined to try to correct the effects of such an absorption by the nitric acid, and to hasten the process of scabbing externally by a wash. It occurred to me that if pure air corrected externally the bad smell from the disease, that the oxygen of the nitric acid would correct the variolous motion internally. The following medicines were now therefore ordered.

Capiat. acid. nitric. ʒj. ex decoct. hord. ℔j. partitis vicibus per diem. Injiciatur vespere enema purgans. Intermittatur decoct. nitros.

18th. Every symptom continues favourable:

Continuatur acidum nitricum; repetatur enema purgans vespere.

19th.

19th. Continuantur medicamenta.

20th. Capiat. acid. nitric. ʒiſs ex decoct. hord. ℔jſs. haust. part. quotidie; repetatur enema purgans vespere.

21st. Continuatur acidum nitricum et capiat cort. peruv. ʒ. ij. magnes. ust. ex aq. menth. ℥jſs bis die.

23d. Continuatur medicamenta.

He now was encrusted with *one scab* over every part of his face and body, but free from any complaint; his appetite returned, and he was allowed boiled milk and bread for supper and breakfast, and pudding for dinner; *no secondary fever took place*, and the scabbing process was very rapid, which was perhaps assisted by a wash of the solution of cerussa acetata; he remained in his room until the scab separated, and his strength was so much restored as to enable him to join his ship; his face and skin on every part of his body was left quite smooth, without any visible pits or other marks; he was visited by most of the medical officers of the charity in the different stages of the eruption, and a nurse remained constantly with him. He was very obedient to every direction, and very grateful for his recovery. He continued the nitric acid and the magnesia usta for another week; the nitric acid still to correct any matter absorbed, and the magnesia usta to carry it off by the bowels; after which, more quickly to restore his strength, he took the cinchona twice a day for two weeks, when he was discharged.

I need not mention the common practice of giving wine and other liquors in confluent small-pox; and even in the distinct species, there is a great inclination to do the same. Whatever conviction there is with regard to the good effects of cool air and cleanliness in small-pox and other diseases, still the *stimulating* plan of diet and medicine creeps in. On this subject, I dare say, many will be desirous to know the practice in the Small-pox Hospital, now under the care of Dr. Adams, and whether it has continued the same for many years. I doubt not but such information, if Dr. Adams will have the goodnes to give it, will be of general use, and it will be particularly acceptable to me; the result of the practice should, however, at the same time be stated.

J. WOOD, M. D.

*Newcastle, April 4, 1806.*

